# Effect of Polyacrylic Acid on Rheology of Cement Paste Plasticized by Polycarboxylate Superplasticizer

**DOI:** 10.3390/ma11071081

**Published:** 2018-06-25

**Authors:** Baoguo Ma, Yi Peng, Hongbo Tan, Zhenghang Lv, Xiufeng Deng

**Affiliations:** State Key Laboratory of Silicate Materials for Architectures, Wuhan University of Technology, Wuhan 430070, China; mbgjob@163.com (B.M.); pengyi@whut.edu.cn (Y.P.); lvzhenghang@whut.edu.cn (Z.L.); dxf@whut.edu.cn (X.D.)

**Keywords:** polyacrylic acid, fluidity, rheology, adsorption, combination

## Abstract

Viscosity-enhancing agents (VEA) have been widely employed in high flowability cement-based materials, so as to ensure that no bleeding and segregation would occur. However, in most cases, interaction between VEA and superplasticizer would be unavoidable. In this study, the effect of polyacrylic acid (PAA), known as one of the most commonly used VEAs, on rheology performance of cement paste containing polycarboxylate superplasticizer (PCE), was studied. The initial fluidity was assessed with mini slump, and rheological behavior of cement paste was evaluated with rotor rheometer. Adsorption amount was examined with total organic carbon (TOC) analyzer, and the zeta potential was also tested. The interaction between PAA and PCE in the presence of calcium ion (Ca^2+^) was analyzed with conductivity, X-ray photoelectron spectroscope (XPS), and dynamic light scattering (DLS). The results illustrate that PAA can adsorb onto the surface of cement particles to plasticize cement paste, being similar to PCE. In the presence of Ca^2+^, PAA can be curled and crosslinked, as a result of the combination between carboxyl groups (COO^−^) and Ca^2+^, thereby affecting the adsorption performance and conformation behavior. It is interesting that negative impact of PAA on dispersion efficiency of PCE can be demonstrated; one reason is the reduced adsorption amount of PCE by PAA competitively adsorbing onto the cement surface, and another possible reason is the invalided PCE by adsorption of PAA. Additionally, molecular weight of PAA should be considered if being used as VEA in PCE system.

## 1. Introduction

No bleeding and segregation is mandatory in high fluidity cement-based materials, such as self-compacting concrete, self-leveling floor, and grout materials. Generally, this can be obtained with addition of viscosity-enhancing agent (VEA) and superplasticizer [1,2,3]. Polyacrylic acid (PAA) is one of the most commonly used viscosity-enhancing agents with an excellent effect on resisting bleeding and segregation [4,5]. The primary mechanism behind includes two aspects: one is that it can increase viscosity of liquid phase by means of the formation of intermolecular network structure, and the other is that PAA molecule with great molecular weight can connect these cement particles through adsorbing on the surface of cement particles [6,7].

Polycarboxylate superplasticizer (PCE), regarded as a kind of high-efficiency water-reducing agent, are used to efficiently plasticize cement paste for achieving high fluidity of cement-based materials by using less mass of water [8,9,10]. The dispersion efficiency of PCE is closely related to the activity of long side chain (PEO, polyethylene oxide, which offers the steric hindrance as the main dispersion force) and the effective adsorption of PCE is the precondition for the effect of long side chain PEO [11]. Generally, greater adsorption amount and higher activity of PEO would lead to stronger dispersion efficiency [12]. However, adsorption of PCE can be perturbed with addition of additives. Retarders in PCE system, such as sodium tripolyphosphate (STPP), sodium gluconate (SG), borax and sulfate, have an obvious impact on the dispersion capacity of PCE [13,14,15,16]. For example, competitive adsorption between SG and PCE would inevitably occur, which could decline the adsorption amount of PCE, thereby weakening the dispersion efficiency of the mixed system. Furthermore, the activity of long side chain PEO could be weakened, in some cases. Many studies had revealed that the insertion of PEO into the interlayer of montmorillonite would notably weaken the effective activity of PEO, leading to the decline in dispersion efficiency. Moreover, some chemicals can also accelerate the inactivation of PEO in PCE [17,18,19]. For example, the intercalation of PCE can be enhanced by STPP to further invalid the PEO, and in this case, the dispersion efficiency of PCE would be declined immediately [15].

Despite that the addition of PAA can obviously increase the viscosity to solve the problems of segregation and bleeding, the negative impact of PAA on dispersion capacity of PCE cannot be ignored. On account of the adsorption of PAA onto the surface of cement particles, the interaction between PAA and PCE must be considered. In order to verify these, the effect of PAA on fluidity and rheology of cement paste in the presence of PCE was investigated in this paper, and the molecular weight of PAA was taken into account. Competitive adsorption between PAA and PCE was analyzed, and the conformation behavior of PAA and PCE was discussed. Finally, a dispersion mechanism was systematically analyzed. These conclusions would be expected to offer useful experience for the use of viscosity-enhancing agent in concrete engineering.

## 2. Materials and Test Methods

### 2.1. Materials

#### 2.1.1. Cement

Cement used in this research is an ordinary portland cement (42.5 Wuhan Yadong Cement Co., Ltd., Wuhan, China), and the fundamental performance of cement is shown in Table 1.

#### 2.1.2. Additives

**Figure 1:**
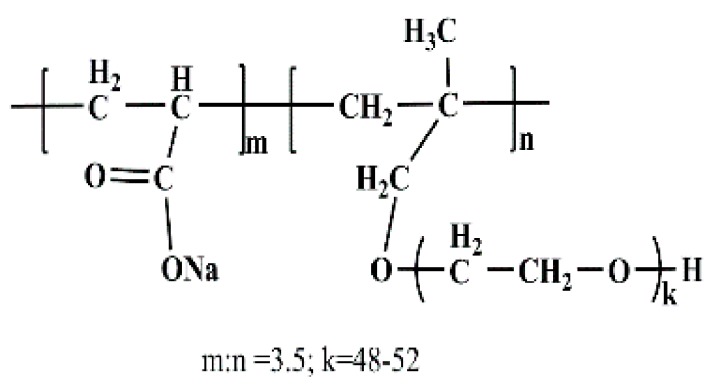
Molecular structure of PCE.

**Figure 2:**
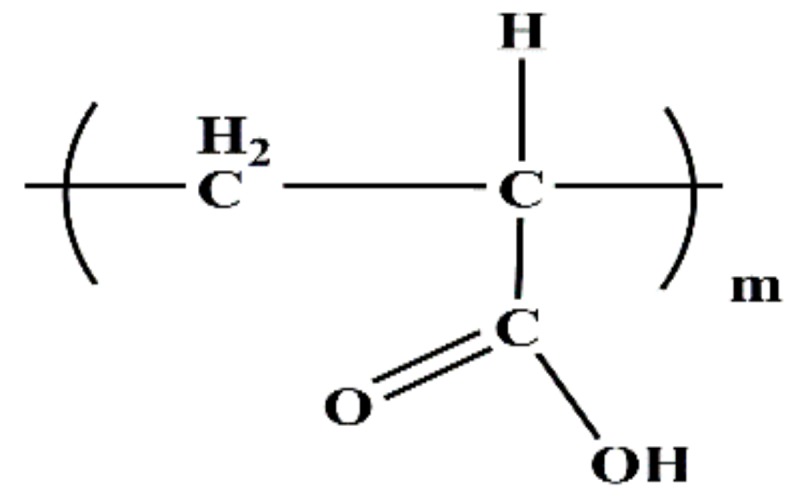
Molecular structure of PAA.

**Figure 3:**
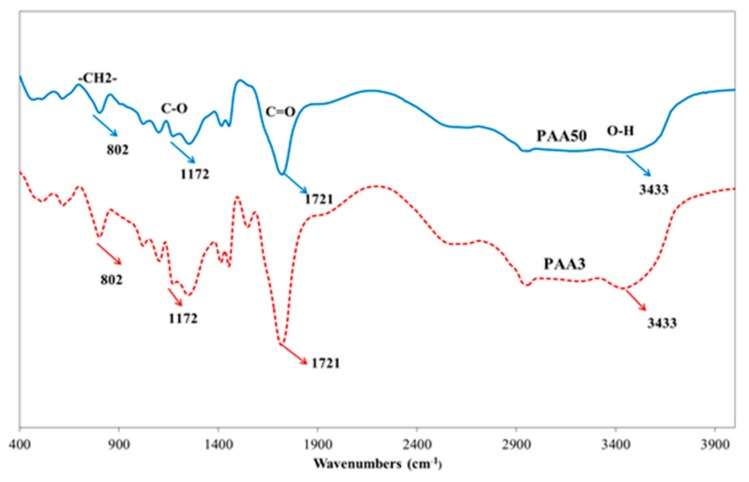
FTIR spectra of PAA.

A commercially available polycarboxylate superplasticizer (PCE, made by Wuhan Huaxuan Co., Ltd., Wuhan, China) was used in this study. The molecular structure and fundamental performance, obtained from the company, are shown in Figure 1 and Table 2. Polyacrylic acid (PAA, made by Shanhai Macklin Biochemical Co., Ltd., Shanghai, China) was employed in this research, and the added dosage was recorded as solid content. The molecular structure and fundamental performance, obtained from the company, are shown in Figure 2 and Table 3. Furthermore, the polymers were dried in a vacuum drying oven at about 65 °C, and then were characterized by Fourier-transform infrared spectroscopy (FTIR, Nexus, made by Thermo Nicolet, Madison, WI, USA). The presence of fundamental chemical groups in PAA could be found from the FTIR spectroscopy. As shown in Figure 3, –CH_2_–, C=O, –OH, and C–O–C groups can be obviously found. This result demonstrates the presence of COO^−^ group in PAA.

### 2.2. Test Methods

#### 2.2.1. Fluidity Performance

Chemicals were mixed with water in advance. Cement paste with PAA (0.00%, 0.04%, 0.08%, 0.12%, 0.16%, 0.20% of cement) were prepared with water and cement ratio (W/C) of 0.5. Cement paste with PAA (0.00%, 0.02%, 0.04%, 0.08%, 0.12%, 0.16%, 0.20% of cement) and PCE (0.1% of cement) were prepared with W/C of 0.29. The initial fluidity (within 5 min) was measured with a truncated cone mold (height: 60 mm; top diameter: 36 mm; bottom diameter: 60 mm) in accordance with the Chinese standard of GB/T 8077-2012. The truncated cone was firstly put on a smooth glass plate, and then it was filled with the cement paste; the cone was slowly lifted vertically, and the maximum diameter of the spread sample and the maximum width perpendicular to that diameter were measured after 30 s. The average of these two values was defined as the initial fluidity value (mm).

#### 2.2.2. Rheology Performance

Cement paste with PAA, and cement paste with PAA and PCE, were prepared with the same process and parameters as above, except that the dosage of PCE in cement with PAA and PCE system is 0.07% of cement, rather than 0.1%. The purpose of reducing the dosage of PCE is to control the yield stress to above zero. These cement pastes were stirred for 5 min, and then, the rotor rheometer (R/S-SST, rotor: CC45, made by Brookfield, New York, NY, USA) was used to test the rheology performance of paste. The process of measurement was divided into four steps. In order to bring the cement paste to a reference structural state, it was firstly pre-sheared at a shear rate equal to 100 s^−1^ for 30 s, and then paused for 10 s. An increasing shear rate was directly applied from 0–120 s^−1^ within 100 s. After that, a decreasing shear rate was applied from 120–0 s^−1^ within 100 s. The Bingham fluid mode was adopted to analyze the data obtained from rheology software (2000 V2.8, Brookfield, NY, USA). Based on this, yield stress and plastic viscosity can be calculated.

#### 2.2.3. Adsorption Amount

**Figure 4:**
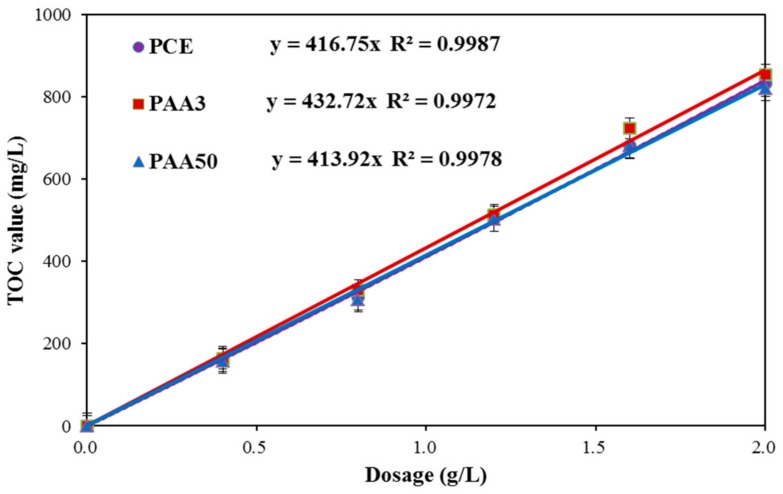
Relation between concentration and total organic carbon (TOC) value.

Total organic carbon (TOC) meter (Elementar, Langenselbold, Germany) was used to measure the adsorption amount of these organics on the surface of cement particles. The carbon content of these organics with various concentrations (0–2.0 g/L) was tested, and the results are shown in Figure 4. This figure indicates the relationship between concentration and carbon content of the polymer, and based on this, the concentration of these polymers can be calculated from the TOC value.

One gram of cement was added into these organics solution with various concentrations (0, 0.4 g/L, 0.8 g/L, 1.2 g/L, 1.6 g/L, 2.0 g/L, 20 mL). Afterwards, these samples were mixed for 5 min, and then centrifuged with high speed centrifugal treatment (HDL-4, Hongke instrument Ltd., Changzhou, China) at 4000 r/min for 5 min. The carbon content in supernatant of these samples was measured. According to Figure 4, the concentration of PCE and PAA in upper supernatant (i.e., residual concentration) was obtained. Adsorption amount of PCE and PAA (mg/g-cement) was calculated as follows:

Adsorption amount = V (C_0_ − C)/m

where C_0_ is the initial concentration (g/L) of PCE and PAA before adsorption; C is the residual concentration (g/L) after adsorption; V is the volume of the solution (mL); and m is the mass of the cement (g).

One gram of cement was also added into the mixed solutions at various concentrations (0.0–8.0 g/L PAA; 1.2 g/L PCE; 20 mL), respectively, the same measuring process as mentioned above was executed. The carbon content of supernatant in PCE–PAA binary system, directly tested by TOC, was defined as the measured results. The summation of the carbon content of supernatant in single system of PAA and PCE after adsorption, which was obtained in the single system, was defined as the expected results. If the expected results were smaller than the measured results, competitive adsorption between PAA and PCE would occur; otherwise, the competitive adsorption would not take place [13].

#### 2.2.4. Zeta Potential

One gram of cement was added into the solutions of PAA at various concentrations (0, 1.0 g/L, 2.0 g/L, 3.0 g/L, 4.0 g/L, 5.0 g/L; 20 mL), respectively, and stirred for 5 min. The cement suspension (1.0 g) was diluted with deionized water (49 g). The Malvern Nano-ZetaSizer instrument (Malvern Instrument Ltd., Malvern, UK) was used to test the zeta potential of the suspension.

#### 2.2.5. Conductivity

Solutions of calcium hydroxide (CH, 1.0 g/L), PAA (4.0 g/L), and PCE (4.0 g/L) were prepared in advance. The electrical conductivity analyzer (Seven Compact S230, Mettler Toledo, Greifensee, Switzerland) was used to measure the conductivity value of these solutions (100 g) or the reference (i.e., deionized water, 100 g) with continuous and even drop of CH solution (300 g), and the data was recorded automatically by the instrument.

#### 2.2.6. Binding Energy of Ca^2+^

Organics solutions (PAA and PCE, 10.0 g/L) were mixed with CH (1.0 g/L), respectively. These samples were dried in a vacuum drying oven, and the temperature was controlled at about 65 °C. Afterwards, these solids were examined with the X-ray photoelectron spectroscopy (XPS, Escalab 250Xi, Thermo Fisher Scientific, Waltham, MA, USA). For the instrument, aluminum is used as an anode target (hv = 1486.6 eV); energy resolution was 0.100 eV. The tested data was processed with XPS Peak Fitting Program (Version 4.0, The Chinese University of Hong Kong, Hong Kong, China), and extra peaks, except the peak belonging to CH, were added to fit the observed curve.

#### 2.2.7. Conformation Behavior of PAA and PCE

Solutions of PAA (2.0 g/L), PCE (2.0 g/L), and PAA-PCE (2.0 g/L PAA, 2.0 g/L PCE) were obtained in advance, and these samples (10.0 g) were added into CH solution (1.0 g/L, 10.0 g) or deionized water (10.0 g), respectively. Afterwards, the dynamic light scattering (DLS, Zetasizer Nano, Malvern instrument Ltd., Malvern, UK) was applied to measure the particle size distribution of the samples.

## 3. Results and Discussion

### 3.1. Fluidity

**Figure 5:**
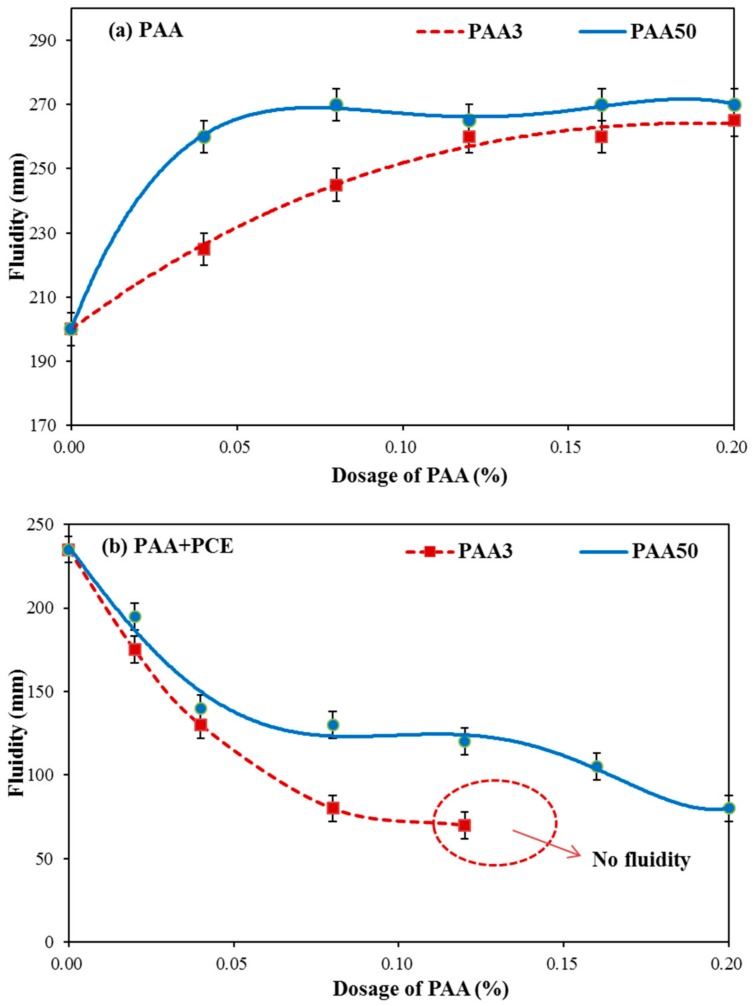
Effect of polymers on fluidity performance of cement paste (**a**: PAA; **b**: PAA–PCE).

Fluidity of cement paste with PAA was measured. As presented in Figure 5a, the fluidity is increased with increasing dosage of PAA, which illustrates that PAA can plasticize the cement paste. By contrast, PAA50 can exert sharper increasing tendency than that of PAA3. This result indicates the stronger plasticizing effect of PAA50 than PAA3.

The fluidity of cement paste in the presence of PAA and PCE (0.10%) was measured. As presented in Figure 5b, the fluidity of cement paste declined with the increasing dosage of PAA3 and PAA50. This result indicates that PAA can decline dispersion capacity of PCE. That is to say, a negative impact of PAA on fluidity of cement paste plasticized by PCE can be observed, which is contrary to the result as presented in Figure 5a. Furthermore, PAA3–PCE system has sharper declining tendency than PAA50–PCE system, indicating that PAA3 has stronger negative impact than that of PAA50.

On the basis of the analysis above, the plasticizing effect of PAA can be found obviously, and possibly, this is associated with the adsorption performance of PAA. Furthermore, PAA has obviously negative impact on flowability of cement paste in the presence of PCE, and negative impact of PAA3 seems stronger than that of PAA50. Probably, the reason is related to the activity of PEO, adsorption amount of PCE, and molecular weight of PAA, as well [3,15].

### 3.2. Rheology Performance

**Figure 6:**
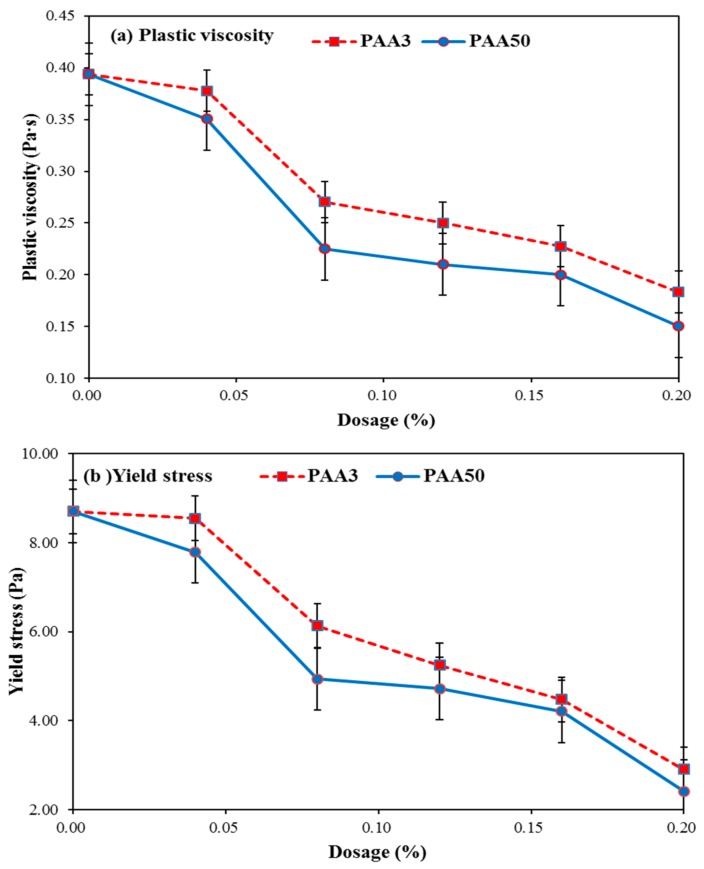
Effect of PAA on rheology performance of cement paste (**a**: plastic viscosity; **b**: yield stress).

In order to further illustrate the negative impact of PAA on the dispersion capacity of PCE, the rheology performance of cement paste with PAA was firstly discussed. As presented in Figure 6a,b, with the increasing dosage of PAA, an obvious declining tendency for the yield stress and plastic viscosity can be seen, which also indicates that PAA can plasticize the cement paste, in agreement with the results as shown in Figure 5a. Furthermore, PAA50 can lead to a sharper declining tendency of the yield stress and plastic viscosity than that of PAA3. That is to say, when the same dosage of PAA was added, the yield stress and plastic viscosity of cement paste with PAA50 is smaller than that of PAA3. These results further demonstrate that PAA50 has stronger plasticizing effect on cement paste than that of PAA3.

**Figure 7:**
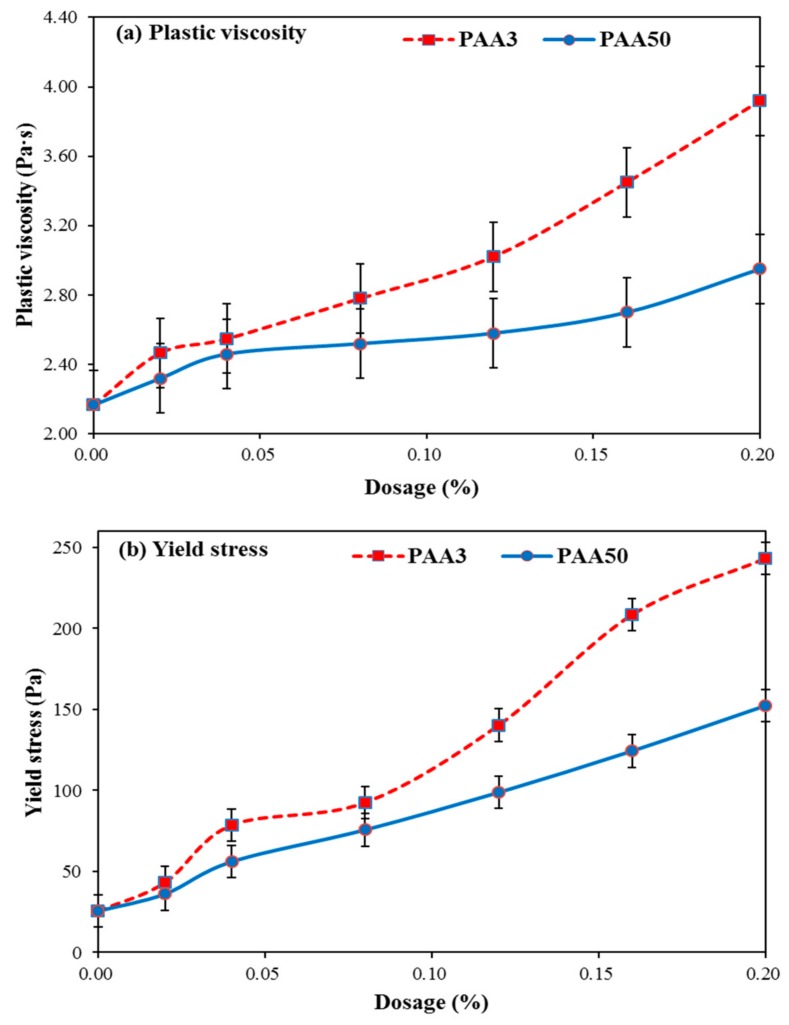
Effect of PAA on rheology of cement paste in the presence of PCE (**a**: plastic viscosity; **b**: yield stress).

The rheology of cement paste with addition of PAA and PCE (0.07%) was discussed. As presented in Figure 7a,b, with the increasing dosage of PAA, yield stress and plastic viscosity are obviously increased. These results also demonstrate that the addition of PAA can lead to an obvious impact on rheology performance of paste in the presence of PCE. A significant reason can be explained in that PAA has a negative impact on dispersion efficiency of PCE.

Based on discussion above, by contrast, PAA3 can make sharper increasing tendency of yield stress and plastic viscosity than that of PAA50, which can be explained by that PAA3 has a smaller plasticizing effect on cement paste than that of PAA50. A stronger negative impact of PAA3 on dispersion capacity of PCE than that of PAA50 can be found, which can be further demonstrated later.

### 3.3. Adsorption Amount

(1) Adsorption Behavior of PAA and PCE

**Figure 8:**
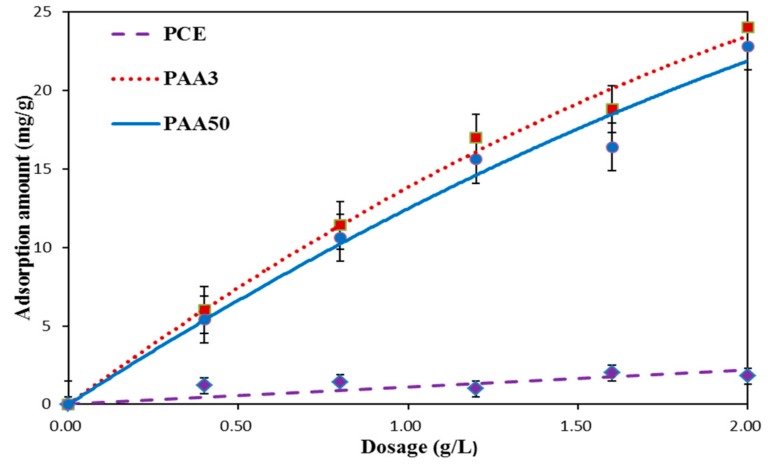
Adsorption amount of PAA and PCE.

Adsorption amount of these organics was presented in Figure 8. An obvious phenomenon can be seen that surface-active points of cement particles can be adsorbed by both PAA and PCE. Whereas, with the identical dosage, the adsorption amount of PAA3 is greater than PAA50, and adsorption amount of PCE is the lowest.

It has been revealed that adsorption of PCE onto the surface of cement particles is the precondition for exerting high dispersion efficiency of PCE [3,20]. Carboxyl groups exist in the side chain of PAA, and these active groups can adsorb on the surface of cement particles by means of chemical combination and electrostatic force [21,22,23]. In this case, mutual influence on adsorption of PAA and PCE, namely competitive adsorption, may take place. This means that many effective adsorption points occupied by PAA cannot be available for PCE.

(2) Competitive Adsorption between PAA and PCE

**Figure 9:**
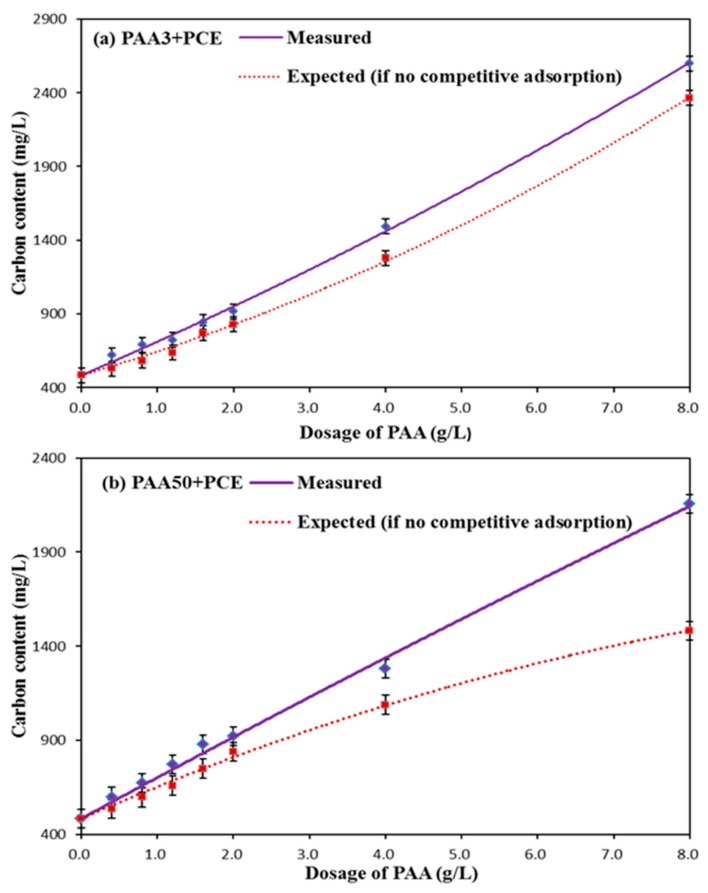
Competitive adsorption between PAA and PCE (1.2 g/L) (**a**: PAA3–PCE; **b**: PAA50–PCE).

To further prove this result, the expected results and the measured results were compared as follows: the expected results are defined as the summation of remainder carbon content in supernatant of single system of PCE and PAA after adsorption with the same dosage, and the measured results, obtained directly from the instrument, are the carbon contents in the supernatant of the PAA–PCE system after adsorption. If there is no obvious difference between these two results, no competitive adsorption would occur; if the expected result were smaller than the measured result, competitive adsorption would happen, indicating that the adsorption of PCE would be perturbed by PAA [12]. As presented in Figure 9, the gap between these two can be observed, which demonstrates the competitive adsorption between PCE and PAA. Accordingly, the addition of PAA would inevitably hinder the adsorption of PCE, thereby resulting in descend on dispersion ability of PCE.

Additionally, the adsorption ability of PAA3 is stronger than PAA50, which means that the competitive adsorption ability of PAA3 is stronger than PAA50. Accordingly, PAA3 can exert stronger negative impact on the fluidity plasticized by PCE, following that the yield stress in PAA3–PCE has sharper increasing tendency than PAA50–PCE. The possible reason for this phenomenon is due to the difference of molecular weight.

### 3.4. Zeta Potential

**Figure 10:**
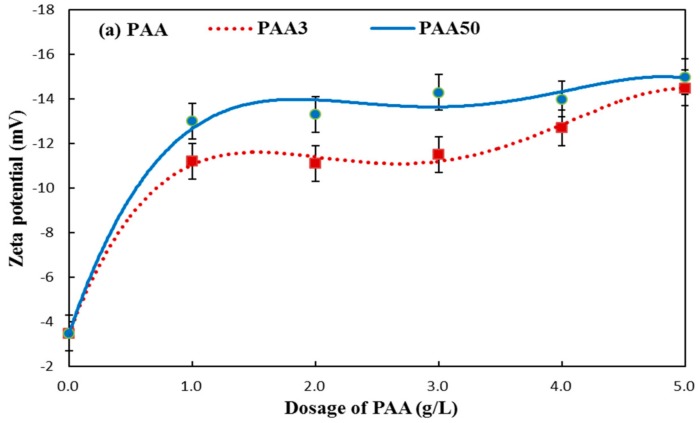
Zeta potential of cement suspension in the presence of PAA.

The relative value of zeta potential depends on the adsorption amount of chemicals in some extent [24,25,26]. The zeta potential of cement suspension with PAA was tested. As presented in Figure 10, the zeta potential is increased with the increasing concentration of PAA. Generally, the change of zeta potential is commonly associated with the adsorption behavior of PAA. By contrast, PAA50 would exert stronger increasing tendency than that of PAA3, as presented in Figure 10, and this could be one of main reasons why PAA50 exerts stronger plasticizing effect than PAA3 (as presented in Figure 5a). However, PAA3 with more adsorption amount should exert more, rather than less zeta potential, and this contradictory result may be related to the difference in adsorption and conformation behavior of PAA, caused by the difference in molecular weight. Especially, PAA causes cement particles to carry a negative charge, and this would interfere with the adsorption of PCE on the surface of cement via electrostatic forces.

### 3.5. Combination of Carboxyl Groups with Ca^2+^

**Figure 11:**
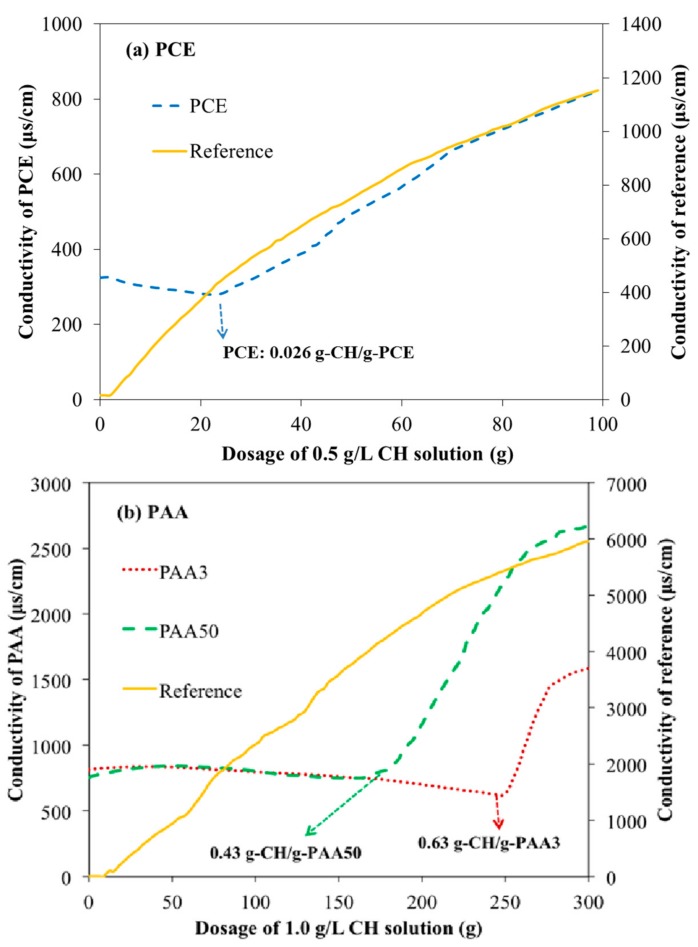
Conductivity of polymers with addition of CH solution (**a**: PCE; **b**: PAA).

Chemical combination with calcium ion (Ca^2+^) as one of the forces, makes polymers adsorb on the surface of cement particle, because these Ca^2+^ molecules are easy to react with carboxyl groups [27,28]. The interaction among these organics and Ca^2+^ would be unavoidable, and this can be illustrated from the conductivity. As presented in Figure 11, with addition of CH (1.0 g/L), the conductivity value of reference has a rapid increase. However, the conductivity value of these polymer solutions is firstly decreased and then increased. This result means that the ions (i.e., Ca^2+^) can be consumed in a range of dosage. When the dosage of CH (0.5 g/L) is increased continuously, the curve of PCE appears a visible turning point, as presented in Figure 11a; and as presented in Figure 11b, other curves of PAA3 or PAA50 also present the same tendency in the presence of CH (1.0 g/L), and these results demonstrate that the combination of Ca^2+^ with these polymers happens before the turning point. That is to say, it can be inferred that at the turning point, almost all available combining chemical groups (i.e., COO^−^) have been consumed. Moreover, the consumption amount of CH solution for PAA3 is 252 g, which indicates that 1.0 g PAA3 would consume 0.63 g CH. Furthermore, for PAA50 it is approximately 180 g, which also indicates that 1.0 g PAA50 would consume 0.43 g CH. This means that PAA3 has a greater combination ability with Ca^2+^ than that of PAA50, which demonstrates the greater adsorption ability of PAA3 than PAA50, in agreement with the adsorption results.

**Figure 12:**
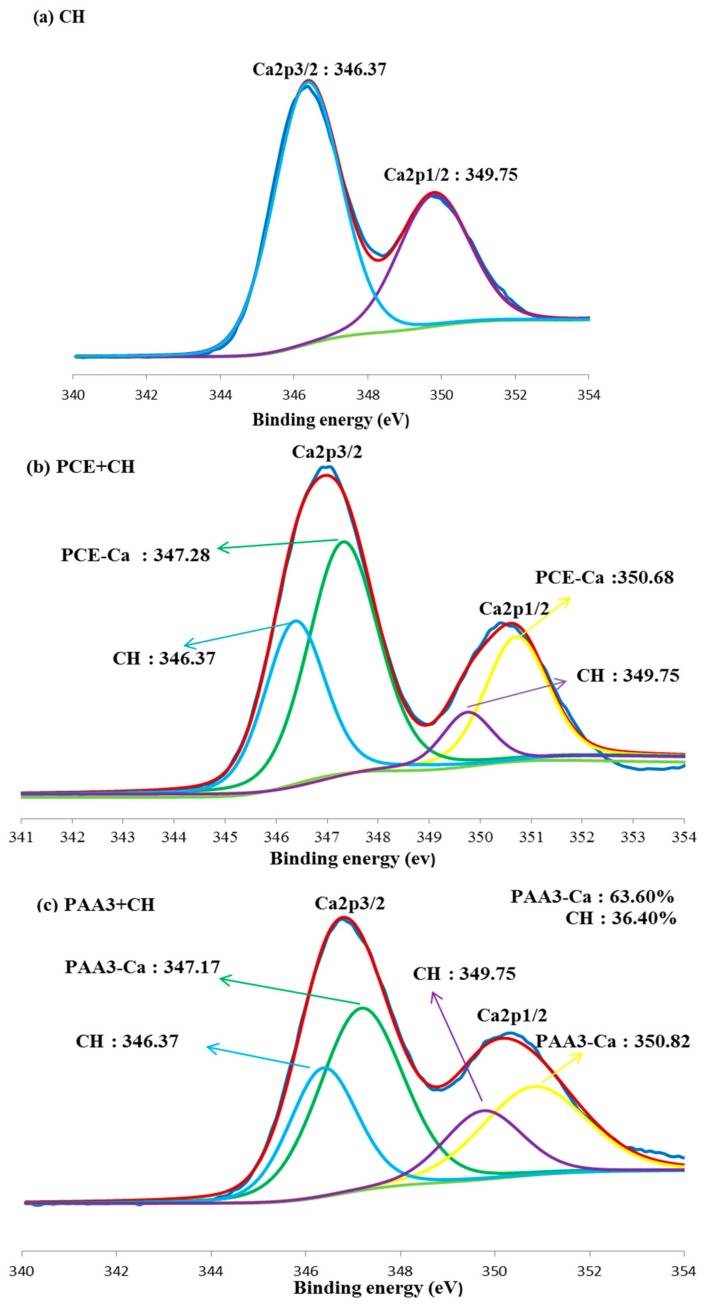

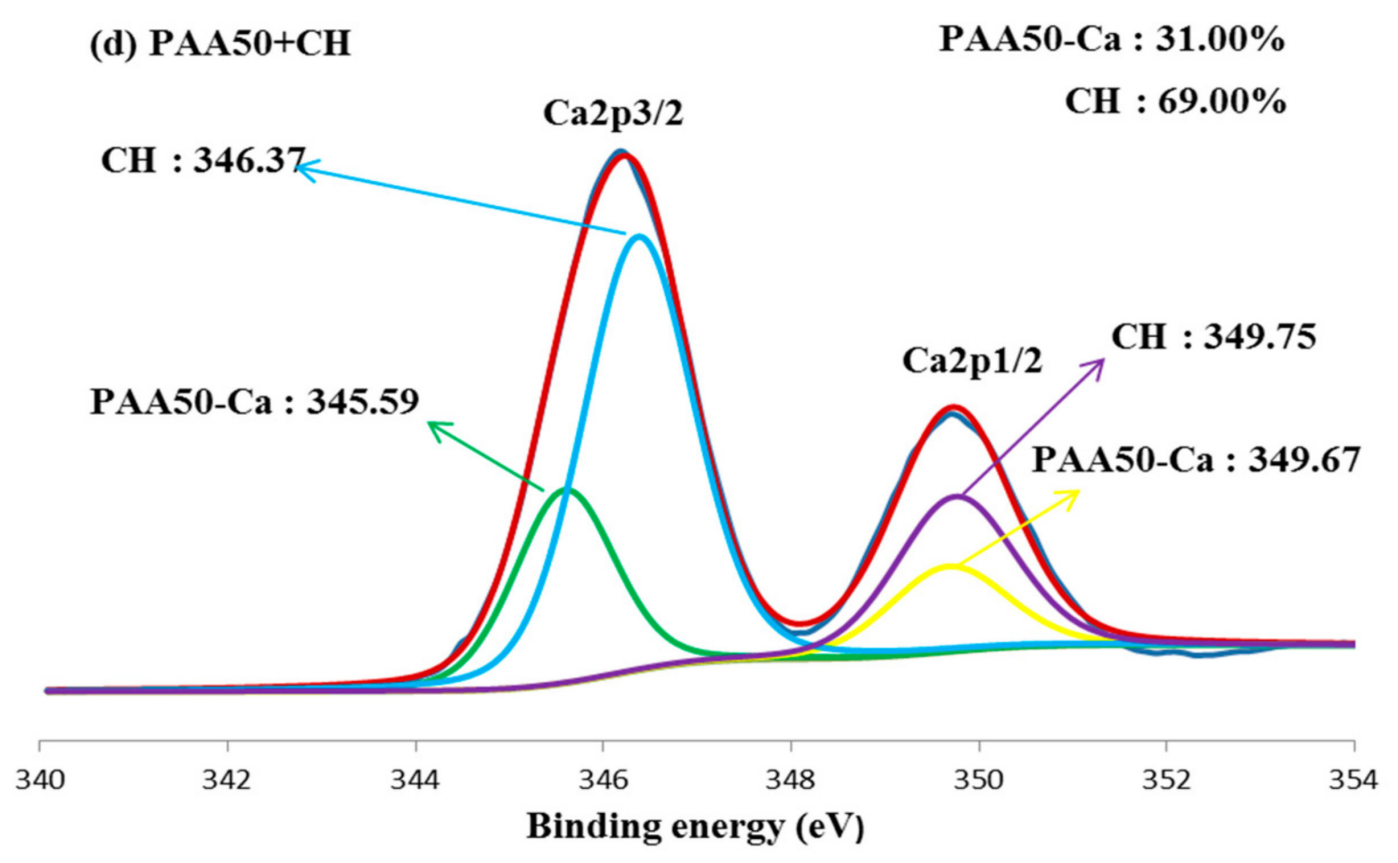
Binding energy of Ca^2+^ (**a**: CH; **b**: PCE-Ca; **c**: PAA3-Ca; **d**: PAA50-Ca).

In order to clarify the reaction of carboxyl groups with Ca^2+^, the binding energy of Ca^2+^ with polymers was characterized with XPS. As presented in Figure 12a, in CH system, the peaks of curve for Ca2p_3/2_ and Ca2p_1/2_ are 346.37 eV and 349.75 eV. Nevertheless, for PCE–CH system, two new peaks can be found, including 347.28 eV for Ca2p_3/2_ and 350.68 eV for Ca2p_1/2_, as presented in Figure 12b. This result indicates that combination of Ca^2+^ with carboxyl groups can occur, and the combination of PCE–Ca in PCE–CH system can be confirmed. It is noticed that the same results have happened in Figure 12c,d. Therefore, these results further illustrate that a kind of new calcium bond, such as PAA3–Ca or PAA50–Ca, has been produced. Additionally, the ratio of these calcium bonds can be calculated from the ratio of each divided peak area. The relative ratio for calcium in PAA–Ca and CH can be obtained according to peak area. As presented in Figure 12c,d, the relative ratio of PAA50–Ca in PAA50–CH is 31.00%, which is much smaller than that of PAA3–Ca in PAA3–CH (63.60%), indicating that PAA3 has stronger combination ability with Ca^2+^ than that of PAA50.

In summary, one crucial conclusion can be drawn that PAA3 has stronger combination capacity with Ca^2+^ than that of PAA50, which is as one of the main reasons for the stronger adsorption ability of PAA3 than PAA50.

### 3.6. Aggregation Behavior of PAA and PCE

**Figure 13:**
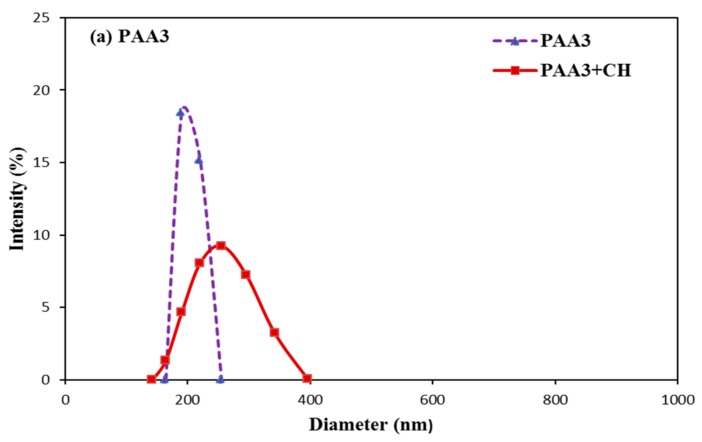

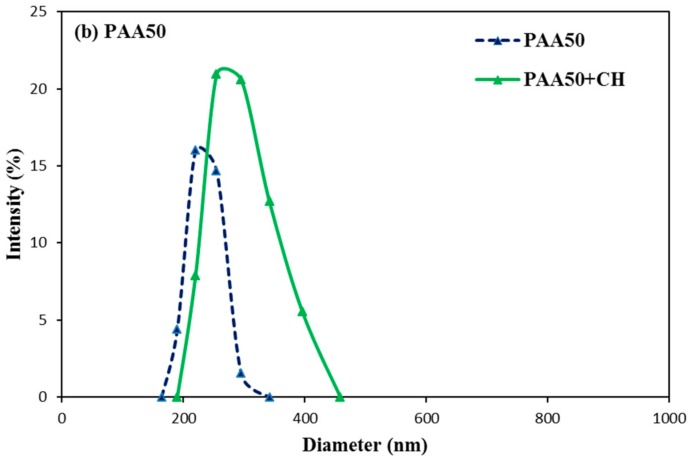
Size distribution of PAA in deionized water and CH solution (**a**: PAA3; **b**: PAA50).

Many studies demonstrated that the conformation of polymers could be altered in the presence of some salts [29,30]. Once cement particles contacted with water, the cement particles would rapidly release CH solution into pore solution [31], the conformation of these polymers could be obviously affected by Ca^2+^ [32]. The size distribution of these polymers was obtained with DLS so as to reveal the conformation behavior of PCE and PAA in the presence and absence of CH. As presented in Figure 13, the size distribution of PAA3 is 164–255 nm, while for PAA50 it is 164–341 nm. With the addition of CH into polymer solutions, the size distribution of polymers for PAA3 and PAA50 is about 161–396 nm and 191–458 nm. In the presence of Ca^2+^, PAA molecule might be curled because of the combination of Ca^2^ and the formation of hydrogen bond; it could be deduced that several carboxyl groups (COO^−^) from different PAA molecules could be connected with one Ca^2+^, and several carboxyl groups (COO^−^) in one PAA molecule could also be connected with one Ca^2+^ [4,5]. In this case, the conformation behavior of PAA would be altered in cement suspension. This result indicates that Ca^2+^ can result in agglomeration of the PAA. Accordingly, PAA can be connected via Ca^2+^, which means that these Ca^2+^ can connect several PAA molecules together.

**Figure 14:**
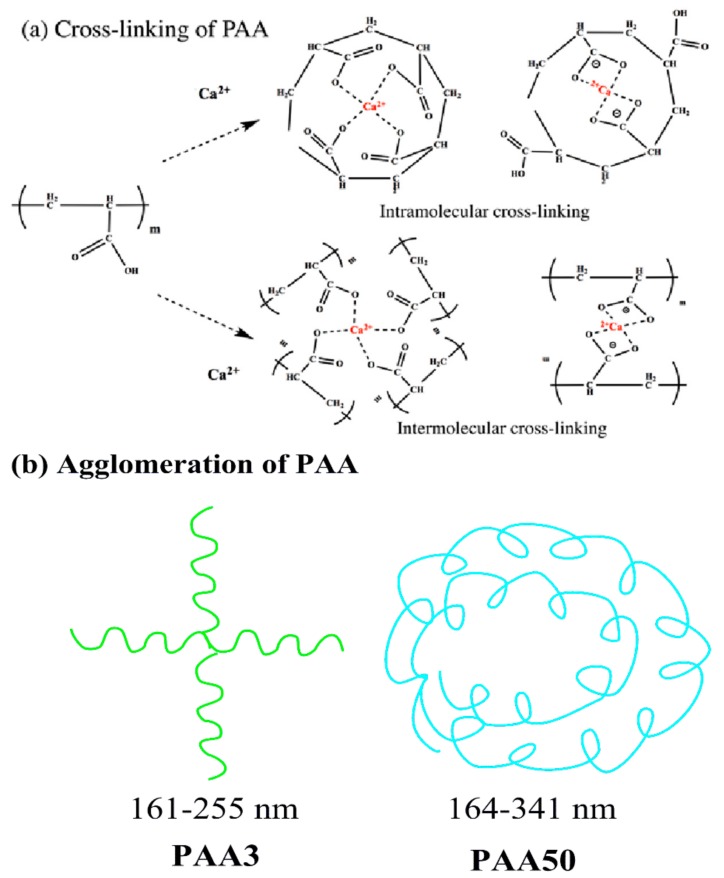
Conformation behavior of PAA in calcium solution (**a**: Cross-linking structures; **b**: agglomeration).

As presented in Figure 14a, the aggregation of PAA3 is probably due to the intermolecular crosslinking, and that for PAA50 is the intramolecular crosslinking. Accordingly, even if there is a much greater molecular weight of PAA50 than that of PAA3, the diameter of PAA50 is only slightly larger than that of PAA3, due to the different curled structures, as presented in Figure 14b.

**Figure 15:**
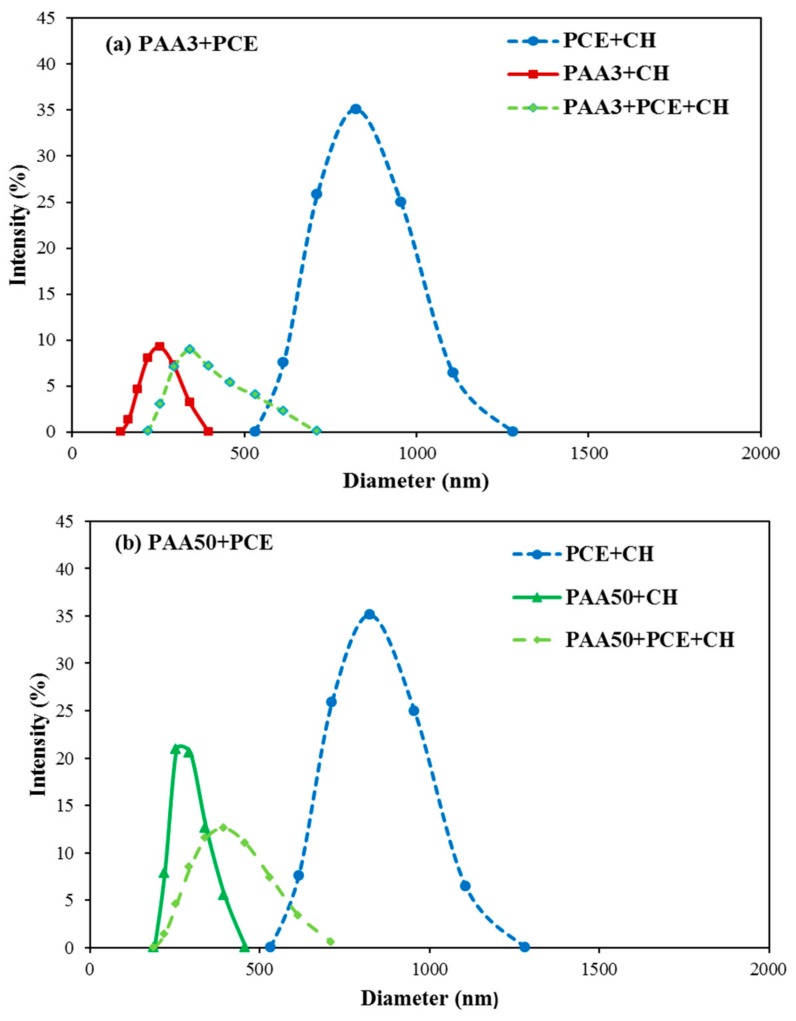
Size distribution of PAA and PCE in CH solution (**a**: PAA3–PCE; **b**: PAA50–PCE).

The size distribution of PCE and PAA system in the presence of CH solution (1.0 g/L) was also measured by DLS. As presented in Figure 15a, in the presence of CH, the size distribution of PCE, PAA3, and PCE–PAA3 is 531–1281 nm, 141–396 nm, and 220–712 nm. If there is no combination between PAA3 and PCE via Ca^2+^, the size of PAA3–PCE would range from 141–1281 nm, or divided into two parts, such as 141–396 nm and 531–1281 nm. However, one obvious phenomenon can be found in the size of PCE–PAA3–CH (i.e., 220–712 nm), which is in the middle of PCE–CH and PAA3–CH. Obviously, greater particles, which should be formed by PCE and CH, disappear. The PAA50–PCE system also shows the same phenomenon, as presented in Figure 15b. This can be probably accounted for by the fact that the addition of PAA into PCE–CH solution can detach the structure of PCE–Ca–PCE, thereby forming a new structure with smaller size (PCE–Ca–PAA). These results indicate the interaction among these polymers via Ca^2+^, which would provide further evidence to prove the combination between PAA and PCE via bridging Ca^2+^.

In this situation, PCE may adsorb on the surface of crosslinking structure of PAA, rather than on the surface of cement particles by combination of Ca^2+^, thereby leading to more amount of ineffective adsorption of PCE [3].

### 3.7. Mechanism

(1) Effect of PAA on fluidity performance of cement paste

**Figure 16:**
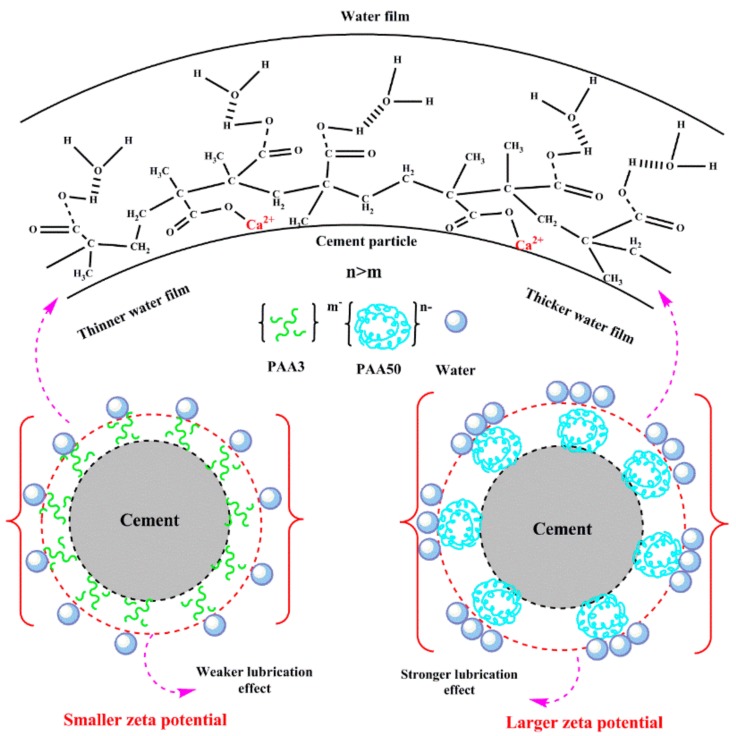
Mechanism model of cement suspension without PCE.

It has been demonstrated that the conformation of PAA can be altered in CH solution, resulting from intramolecular crosslinking and intermolecular crosslinking, as presented in Figure 15. In cement suspension, as presented in Figure 16, PAA molecule can adsorb onto the surface of cement particles to form water film via hydrogen-bond effect of hydrophilic group, thereby lubricating cement particles to improve the fluidity. Furthermore, PAA also can increase the zeta potential of cement particles to offer dispersion force, thereby increasing the fluidity of cement paste. Due to the plasticizing effect of PAA, the yield stress and plastic viscosity of the paste can decline with the addition of PAA.

The main reason for stronger plasticizing effect of PAA50 than that of PAA3 can be illustrated as follows: on the one hand, because the molecular weight of PAA50 is much greater than that of PAA3, and the curled degree of PAA50 in Ca^2+^ ions solution is much stronger than that of PAA3, probably forming the spherical structure with nano particles. These spherical structures can adsorb onto the surface of cement particles and exert stronger lubricating effect to plasticize the cement paste. On the other hand, the aggregation of PAA50 can wrap lots of carboxyl groups into the inside of the spherical structure, resulting in larger negative charge than that of PAA3. In this case, PAA50 adsorbed on the surface of cement particles can bring out stronger zeta potential to plasticize the cement paste.

(2) Effect of PAA on performance of PCE

Addition of PAA into PCE can significantly reduce the dispersion capacity of the system, and the main reason includes three aspects, as follows:

First of all, on account of the competitive adsorption among these organics, adsorption points would be consumed by PAA molecule, and these occupied active points cannot be available for PCE [16,33]. The adsorption of PAA would perturb adsorption of PCE, and the effective adsorption of PCE would be decreased, thereby weakening the dispersion efficiency of PCE.

**Figure 17:**
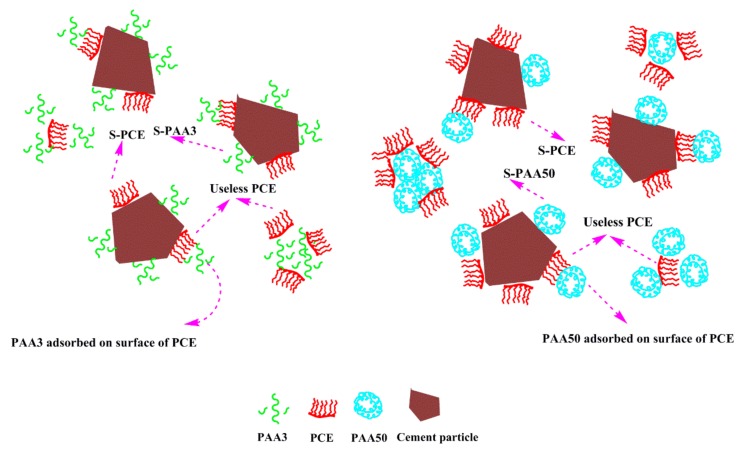
Mechanism model of cement suspension with PCE.

Moreover, as presented in Figure 17, similar to PAA, PCE can also adsorb onto the surface of these crosslinking structures, or be fixed inside the structure via combining Ca^2+^. Therefore, the adsorption amount of PCE on the surface of cement particles can decline, thereby weakening the dispersion ability of PCE. Moreover, PCE adsorbed on the surface of cement particles would be covered by the crosslinking structure of PAA, which can weaken the activity of PEO and decrease the dispersion efficiency of PCE [34,35,36].

However, the negative impact of PAA3 and PAA50 can be distinguished clearly, as shown in the following:

Since PAA3 has stronger adsorption capacity than that of PAA50, the competitive adsorption ability of PAA3 is stronger than that of PAA50, which means that PAA3 would exert larger obstruction to the adsorption of PCE. This result indicates that PAA3 would result in a stronger ability to decline the dispersion of the system. Furthermore, zeta potential caused by PAA50 is stronger than that of PAA3, and the lubricating effect of PAA50 is also stronger; this implies that PAA50 has a stronger ability to contribute to the fluidity. These aspects can be related to the reason why the fluidity of cement paste containing PCE-PAA50 is greater than that of PCE-PAA3. It can be deduced that the competitive adsorption effect between PCE and PAA3 should have a predominant role in causing sharper decline in fluidity and quicker increase in plastic viscosity and yield stress (as presented in Figure 7b).

In view of these points, mentioned above, the negative impact of PAA on dispersion ability of PCE should be taken into account when PAA is used as additive. In addition, the molecular weight of PAA, as one vital factor, should be considered so as to mitigate the negative impact as much as possible. It is suggested that PAA with greater molecular weight would be more suitable to solve the problems of bleeding and segregation in concrete engineering, with less negative impact on the plasticizing effect of superplasticizer.

## 4. Conclusions

(1) PAA can adsorb onto the surface of cement particles, and smaller molecular weight of PAA has stronger adsorption ability. In the presence of Ca^2+^, PAA can be curled, as a result of the combination between carboxyl groups and Ca^2+^. PAA with greater molecular weight may be curled more obviously, and form a spherical structure in the presence of Ca^2+^.
(2) PAA can plasticize cement paste, because of the lubricating effect and the increased zeta potential; greater molecular weight results in stronger plasticizing ability.
(3) PAA has a negative effect on dispersion of PCE, and smaller molecular weight results in stronger negative effect. The main reason for this is due to the competitive adsorption effect.

## Figures and Tables

**Table 1:** Fundamental performance of cement.

Flexural Strength (MPa)		Compressive Strength (MPa)		Setting Time (min)	
3 d	28 d	3 d	28 d	Initial setting	Final setting
4.6	7.6	25.6	45.5	238	291

**Table 2:** Fundamental performance of PCE.

Cl^−^ (%)	Alkali Content (%)	Water reducing Ratio (%)	pH Value	Solid Content (%)	M_w_ (g/mol)
0.03	3.75	30.1	7.2	40.0	67,500

**Table 3:** The fundamental performance of PAA.

	Solid Content (%)	pH Value	M_w_ (g/mol)
PAA3	30	3.0–3.5	3000
PAA50	50	2.5–3.0	50,000
